# Integrated metabolomic and transcriptomic analysis of the mechanism underlying pericarp variegation in *Citrus reticulata* ‘Gonggan’

**DOI:** 10.3389/fpls.2026.1960602

**Published:** 2026-09-24

**Authors:** Congyi Zhu, Pingzhi Wu, Yibo Hu, Ruimin Zhang, Hongqi Xia, Zhengyan Fan, Pengtao Yue, Chuanhong Ren, Peihua Lin, Sai Xu, Yongjing Huang

**Affiliations:** 1 Institute of Fruit Tree Research, Guangdong Academy of Agricultural Sciences, Key Laboratory of South Subtropical Fruit Biology and Genetic Resource Utilization (MOA), Guangdong Province Key Laboratory of Science and Technology Research on Fruit Tree, Guangzhou, China; 2 School of Life Sciences, Zhaoqing University, Zhaoqing, China; 3 Deqing Citrus Research Institute, Zhaoqing, China; 4 Institute of Facility Agriculture, Guangdong Academy of Agricultural Sciences, Guangzhou, China

**Keywords:** chlorophyll degradation, differential metabolite, differentially expressed genes (DEGs), the variegated ‘Gonggan’ mandarin, transcriptomics

## Abstract

**Introduction:**

Understanding the molecular mechanisms underlying fruit color variation is crucial for citrus breeding. The variegated ‘Gonggan’ mandarin (*Citrus reticulata* Blanco) exhibits distinct phenotypic differences compared to the normal cultivar. However, the physiological and genetic basis for this variation remains unclear.

**Methods:**

In this study, we integrated physiological measurements, liquid chromatography-mass spectrometry (LC-MS), and transcriptome sequencing to characterize the pericarp of the variegated ‘Gonggan’ mandarin during early fruit development stage.

**Results:**

Compared with the normal ‘Gonggan’ mandarin, lightness and chromaticity values of the pericarp color exhibited significantly elevated in the variegated ‘Gonggan’ mandarin. Physiological assays revealed a drastic reduction in chlorophyll a and b contents (by 75.76% and 58.33%, respectively) and a moderate increase in total carotenoids (28.61%) in the variegated ‘Gonggan’ mandarin. Notably, phytoene accumulation in the pericarp of the variegated ‘Gonggan’ mandarin was 44.35-fold higher than that in the normal fruit, while metabolites in the middle and downstream pathway of carotenoid biosynthesis such as β-carotene and lutein were significantly reduced. Transcriptome analysis identified 1,438 differentially expressed genes (DEGs), which were predominantly enriched in metabolic pathways, secondary metabolite biosynthesis and photosynthesis. Specifically, 3 chlorophyll degradation genes encoding chlorophyll b reductase gene, chlorophyllase and SGR protein, 23 photosynthesis genes (*PsaD*, *PsaE*, *PsbO*, *etc*.), and 6 photosynthesis-antenna proteins (*Lhca1*, *Lhca2*, *Lhcb1*, *etc*.) were significantly upregulated. Furthermore, 13 DEGs associated with plant hormone signal transduction exhibited differential expression patterns.

**Discussion:**

Our findings demonstrate that the disruption of the chlorophyll degradation pathway and carotenoid biosynthesis pathway are the primary causes of the pericarp color variation in the variegated ‘Gonggan’ mandarin. This study provides novel insights into the regulatory network governing pericarp coloration and establishes a foundation for future molecular breeding strategies targeting citrus appearance quality.

## 1 Introduction

Citrus fruit is the primary category of tree fruit crops in the world for their organoleptic properties and their nutraceutical value. Fruit color is a major component of external fruit quality and directly influences consumer preference, thereby strongly affecting marketability and economic return (Lado et al., 2019). With increasing consumer demands for superior internal and external fruit quality, breeding novel citrus cultivars with desirable coloration has become an important objective in citrus improvement (Zacarías-García et al., 2022). Color variation in citrus fruit occurs in both the pericarp and pulp. Compared with pericarp color, pulp color variation has been more extensively reported, including ‘Hong Anliu’ sweet orange (Yu et al., 2012; Shi et al., 2018), ‘Cara Cara’ navel orange (De Ancos et al., 2020; Liu et al., 2021), ‘Niurouhong’ kumquat (Tang et al., 2022), and red-fleshed ‘Huazhou’ pomelo (Zhu et al., 2022). Pericarp color is a key determinant of citrus appearance and has a substantial impact on commercial value. Previous studies have shown that citrus pericarp coloration is mainly determined by carotenoids, chlorophylls, and anthocyanins (Li et al., 2021; Zhai et al., 2022; Costanzo et al., 2022). The specific color phenotype results from the differential accumulation of these pigments across cultivars. Typically, unripe citrus fruits exhibit green pericarp due to high chlorophyll content. As fruit development and ripening proceed, chlorophyll degradation coincides with carotenoid accumulation, resulting in yellow or orange pericarp (Huang et al., 2018; Lu et al., 2022).

In recent years, several novel citrus germplasms such as orange-pericarp pomelo (Guo et al., 2015), ‘Qingou gan’ (Zhu et al., 2017), ‘Pinalate’ sweet orange (Rodrigo et al., 2019) red-skinned ‘Huyou’ (Luan et al., 2020) and ‘Zongcheng’ navel orange (Zhu et al., 2021) have attracted considerable consumer attention due to their unique pericarp coloration. Among them, orange-pericarp pomelo, a bud mutant of ‘Guanxi’ pomelo, develops an orange pericarp at maturity, whereas the wild type has a light-yellow pericarp. Analysis of carotenoid composition showed that the total carotenoid and β-carotene content in the pericarp of orange-pericarp pomelo were 7.9-fold and 10.5-fold higher than those in the wild type, respectively, whereas no significant differences were observed in pulp carotenoids (e.g., β-carotene, lycopene) (Guo et al., 2015). Among plant carotenoids, β-citraurin exhibits one of the strongest red color contributions, second only to lycopene. Luan et al. (2020) reported that the total carotenoid content in the pericarp of red-skinned ‘Huyou’ was 1.8-fold higher than that in normal ‘Huyou’, whereas lycopene was not detected in either genotype. In addition, β-citraurin accounted for 7% of the total carotenoids in red-skinned ‘Huyou’ peel but was undetectable in normal ‘Huyou’, suggesting that β-citraurin is a major contributor to the red pericarp phenotype. ‘Zongcheng’ is a bud mutant of ‘Lane Late’ navel orange with a distinctive brown pericarp, although its flesh color is the same as that of the wild type and remains orange. Zhu et al. (2021) attributed this phenotype to impaired chlorophyll degradation coupled with elevated levels of phytoene, phytofluene, and violaxanthin, resulting in a brownish color via the superposition of persistent green and accumulated orange pigments.

The ‘Gonggan’ mandarin (*Citrus reticulata* Blanco var. ‘Gonggan’), a geographically indicated cultivar endemic to Zhaoqing, China, is widely regarded as the ‘king of citrus’ due to its superior organoleptic quality. As a natural hybrid of mandarin and sweet orange, the ‘Gonggan’ mandarin exhibits a unique combination of desirable traits, inheriting the sweet flavor and juicy texture characteristic of sweet oranges, alongside the easy-peeling rind and crisp, tender pulp typical of mandarins (Shi et al., 2025). the variegated ‘Gonggan’ mandarin is a spontaneous branch mutation discovered in Deqing County, Guangdong Province, China. Despite the commercial potential of these variants, the molecular mechanisms underlying their unique pericarp coloration remain unclear. In the present study, we investigated changes in color parameters, chlorophyll content, and carotenoid content in young fruits of the variegated ‘Gonggan’ mandarin to analyze the physiological basis of its color variation. Furthermore, transcriptome sequencing will be employed to identify differentially expressed genes (DEGs), with the aim of providing evidence for a deeper understanding of the mechanism underlying pericarp variegation in ‘Gonggan’ mandarin.

## 2 Materials and methods

### 2.1 Plant materials and sampling

Fruit samples were collected from the fruit tree germplasm resource nursery in Guangdong Province, China. The germplasm resource nursery was located at 113°12′ E, 23°9′ N, with an annual mean temperature of 21.4–21.9 °C and annual precipitation of 1623.6-1899.8 mm. The variegated ‘Gonggan’ mandarin grafted on red tangerine (*C. reticulata* Blanco) was used as the experimental materia. Fruits were harvested at 130 days after full bloom (DAFB). The pericarp sample were excised from the equatorial zone of the variegated ‘Gonggan’ mandarin fruit, ensuring the complete removal of the underlying albedo. The normal ‘Gonggan’ mandarin grown under identical grafting and cultivation conditions were used as the control.

### 2.2 Fruit pericarp color difference analysis

Fruit pericarp color of both variegated and normal ‘Gonggan’ mandarin was measured using an automatic colorimeter (CR-400, Konica Minolta, Japan). The color parameters, including lightness (L*), chroma (C*), hue angle (*h°*), redness (a*), and yellowness coordinate (b*), were evaluated based on the CIELAB color space system. Ten fruits were randomly selected and measured for each sample group.

### 2.3 Determination of photosynthetic pigment contents

Pericarp tissues from the equatorial region of the fruit were collected, frozen in liquid nitrogen, and ground into powder using a tissue homogenizer (T10, IKA, Germany). After rapid weighing, photosynthetic pigments were extracted according to the method of Wellburn and Lichtenthaler (1984) using acetone extraction solution (acetone: absolute ethanol = 1:1, v/v) for 2 h. The absorbance of the extract was measured at 645 and 663 nm with a UV-1800 spectrophotometer (Shimadzu, Japan). Chlorophyll a and chlorophyll b contents in the fruit pericarp were calculated using the equations described by Arnon (1949).

### 2.4 Extraction, identification, and quantification of carotenoids

A total of 50 mg of freeze-dried sample was ground into powder and extracted with 0.5 mL of hexane/acetone/ethanol (1:1:2, v/v/v) containing 0.01% butylated hydroxytoluene (BHT). After concentration, the extract was re-dissolved in 100 μL methanol/methyl tert-butyl ether (3:1, v/v) for chromatographic and mass spectrometric analysis.

Data acquisition was performed using an ultra-performance liquid chromatography system (UPLC, ExionLC™ AD, SHIMADZU, Kyoto, Japan) coupled with a tandem mass spectrometer (MS/MS, Applied Biosystems AB Sciex 6500 Triple Quadrupole, Foster City, CA, USA). Chromatographic separation was achieved on a YMC C30 column (100 mm × 2.0 mm, 3 μm). The mobile phases consisted of solvent A, methanol/acetonitrile (1:3, v/v) containing 0.01% BHT and 0.1% formic acid, and solvent B, methyl tert-butyl ether containing 0.01% BHT. The gradient elution program was as follows: 0 min, 100:0 (A:B, v/v); 3 min, 100:0; 5 min, 30:70; 9 min, 5:95; 10 min, 100:0; and 11 min, 100:0. The flow rate was 0.8 mL min^−1^, the column temperature was maintained at 28 °C, and the injection volume was 2 μL.

Mass spectrometric analysis was conducted using an atmospheric pressure chemical ionization (APCI) source with a source temperature of 350 °C and curtain gas set at 25 psi. Multiple Reaction Monitoring (MRM) mode was utilized for quantification. Standard solutions of individual carotenoids at different concentrations were prepared to generate calibration curves. Carotenoid contents in the samples were quantified based on the integrated peak areas.

### 2.5 RNA extraction, library construction, and transcriptome sequencing

Total RNA was extracted using a plant total RNA extraction kit (Tiangen, Beijing, China). RNA concentration and purity were determined using a NanoDrop 2000 spectrophotometer (Thermo Scientific, USA). Library construction and transcriptome sequencing were performed by Novogene Co., Ltd. (Beijing, China) on the Illumina HiSeq 2500 platform. Raw sequencing data were first evaluated using FastQC, and low-quality reads were removed using Trimmomatic to obtain clean reads. The sweet orange genome (https://www.citrusgenomedb.org/) was used as the reference genome, and read alignment was performed using HISAT2. Transcript abundance in each sample was calculated as fragments per kilobase of transcript per million mapped reads (FPKM) using RSEM. Differentially expressed genes (DEGs) between samples were identified using DESeq2 with thresholds of |log_2_(Fold Change)| > 1 and false discovery rate (FDR) < 0.05. Functional enrichment analyses, including Gene Ontology (GO) enrichment and Kyoto Encyclopedia of Genes and Genomes (KEGG) pathway enrichment analyses of DEGs were performed using Blast2GO and KOBAS, respectively.

### 2.6 Statistical analysis

Three biological replicates were performed for all experiments. All experimental data are presented as means ± standard deviation (SD). Significant differences between the variegated and normal groups were determined using independent samples *t*-tests. Statistical analyses were performed using SPSS 16.0 software (IBM Corp., Armonk, NY, USA), with *p* < 0.05 considered statistically significant.

## 3 Results

### 3.1 Fruit characteristics of variegated ‘Gonggan’ mandarin

The variegated ‘Gonggan’ mandarin was initially identified as a spontaneous bud mutant from the normal ‘Gonggan’ mandarin tree in Deqing County, Guangdong Province, China. After repeated top-grafting identification over multiple years and regional variety trials, it was confirmed as a variegated mutant of ‘Gonggan’ mandarin. Throughout the entire growth period, the leaves of the mutant consistently exhibited a distinct variegated phenotype characterized by the coexistence of green and yellow sectors (Shi et al., 2025). Although the variegated ‘Gonggan’ mandarin was able to flower and bear fruit normally, its growth vigor and yield were slightly lower than those of the normal ‘Gonggan’ mandarin. During the entire fruit development stage, variegated ‘Gonggan’ mandarin fruits displayed an obvious sectorial chimeric phenotype (**Figure 1**). In immature fruit, narrow green longitudinal stripes were interspersed within the yellow peel. Upon maturation, these stripes transitioned to orange against a yellow background. Fruit quality of the variegated ‘Gonggan’ mandarin was comparable to that of normal ‘Gonggan’ mandarin with an edible ratio of 78%, total soluble solids content of 9.0% - 12.5%, total sugar content of 8.5 - 11.0 g/100 g, titratable acidity of 3–5 g/kg, and vitamin C content of 18.8 - 21.9 mg/100 g.

**Figure 1:**
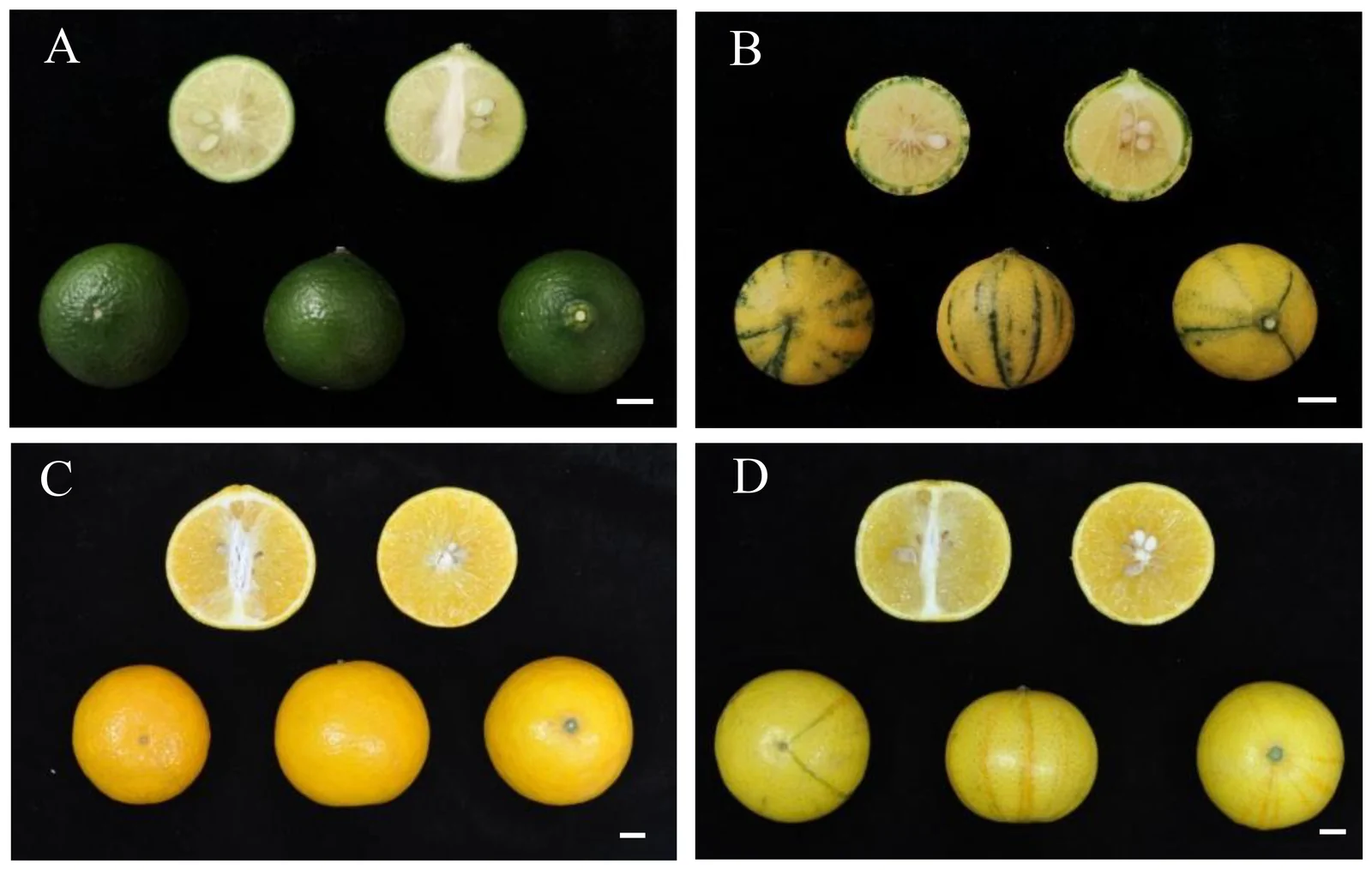
Morphological characters of variegated ‘Gonggan’ mandarin fruit. **(A)**, the young fruit of the normal ‘Gonggan’ mandarin; **(B)**, the young fruit of the variegated ‘Gonggan’ mandarin; **(C)**, the ripe fruit of the normal ‘Gonggan’ mandarin; **(D)**, the ripe fruit of the variegated ‘Gonggan’ mandarin. Bar=10 mm.

### 3.2 Analysis of pericarp color difference, chlorophyll and carotenoid contents in young fruits of variegated ‘Gonggan’ mandarin

In the colorimetric analysis, the L* value represented lightness. The L* value of the young fruit pericarp of variegated ‘Gonggan’ mandarin was 2.20-fold that of normal ‘Gonggan’ mandarin, indicating a marked increase in pericarp brightness (**Table 1**). The a* value reflected the degree of redness relative to greenness, with positive values indicating redness and negative values indicating greenness, and more negative values indicated deeper green coloration. As shown in **Table 1**, the a* values of both variegated and normal ‘Gonggan’ mandarin young fruit pericarp were negative, but the a* value of the former (-4.89 ± 0.44) was significantly higher than that of the latter (-11.46 ± 0.41), indicating that the variegated ‘Gonggan’ mandarin has a lighter pericarp with markedly reduced greenness. The b* value represented the degree of yellowness versus blueness, and a higher b* value indicated a more yellow color. The b* value of variegated ‘Gonggan’ mandarin young fruit pericarp was 3.56-fold that of normal ‘Gonggan’ mandarin, indicating a deeper yellow coloration in the mutant pericarp.

**Table 1:** Analysis of the color parameter and chlorophyll content in the young fruit pericarp of variegated ‘Gonggan’ mandarin.

Sample	L*	a*	b*	Chlorophyll a mg/g	Chlorophyll b mg/g	Chlorophyll a+b mg/g	Chlorophyll a/b
CK	32.19 ± 0.27	-11.46 ± 0.41	14.85 ± 0.53	0.33 ± 0.01	0.12 ± 0.02	0.45 ± 0.01	3.09 ± 0.65
Var	70.94 ± 1.90^*^	-4.89 ± 0.44^*^	52.93 ± 2.63^*^	0.08 ± 0.00^*^	0.05 ± 0.01^*^	0.13 ± 0.01^*^	1.85 ± 0.45^*^

CK, normal ‘Gonggan’ mandarin; Var, variegated ‘Gonggan’ mandarin; Values were expressed as mean ± standard deviation (SD); “*” indicates *p* < 0.05 compared to CK.

Physiological analysis of pigment contents showed that chlorophyll a and chlorophyll b contents in the young fruit pericarp of variegated ‘Gonggan’ mandarin decreased by 75.76% and 58.33%, respectively, compared with those in normal ‘Gonggan’ mandarin, and total chlorophyll content was only 28.89% of that in the control, with extremely significant differences (**Table 1**). In addition, the chlorophyll a/b ratio in variegated ‘Gonggan’ mandarin young fruit pericarp was significantly lower than that in normal ‘Gonggan’ mandarin.

Qualitative and quantitative analyses of carotenoid metabolites in the young fruit pericarp of variegated ‘Gonggan’ mandarin were performed using HPLC-MS/MS, and a total of 27 carotenoids were detected. As shown in **Table 2**, the total carotenoid content in the young fruit pericarp of variegated ‘Gonggan’ mandarin reached 590.44 ± 24.07 μg/g, representing an increase of 28.61% compared with that in normal ‘Gonggan’ mandarin. Notably, phytoene exhibited the most pronounced change. Its content in the young fruit pericarp of variegated ‘Gonggan’ mandarin reached 395.33 ± 8.97 μg/g, which was 44.35-fold that in normal ‘Gonggan’ mandarin. In contrast, the contents of β-carotene, lutein, neoxanthin, violaxanthin, and α-carotene were significantly lower in variegated ‘Gonggan’ mandarin than in normal ‘Gonggan’ mandarin. These results indicated that the mottled pericarp phenotype of variegated ‘Gonggan’ mandarin was closely associated with changes in chlorophyll and carotenoid contents and their relative proportions.

**Table 2:** Analysis of carotenoid content (μg/g DW) in the young fruit pericarp of variegated ‘Gonggan’ mandarin.

Compounds	CK	Var
phytoene	8.91 ± 0.66	395.33 ± 8.97^*^
lutein	342.00 ± 9.45	146.33 ± 12.41^*^
β-carotene	29.33 ± 1.05	9.86 ± 1.05^*^
neoxanthin	25.40 ± 1.64	10.23 ± 1.22^*^
violaxanthin	22.80 ± 0.23	11.17 ± 1.23^*^
α-carotene	16.90 ± 0.35	5.99 ± 0.34^*^
antheraxanthin	2.23 ± 0.15	1.62 ± 0.18
β-cryptoxanthin	2.91 ± 0.17	1.01 ± 0.05^*^
zeaxanthin	1.18 ± 0.05	1.15 ± 0.09
γ-carotene	0.13 ± 0.01	0.16 ± 0.01
lycopene	0.03 ± 0.02	0.05 ± 0.00

CK, normal ‘Gonggan’ mandarin; Var, variegated ‘Gonggan’ mandarin; Values were expressed as mean ± standard deviation (SD); DW, dry weight; “*” indicates *p* < 0.05 compared to CK.

### 3.3 Differential gene expression analysis

#### 3.3.1 Quality analysis of transcriptome data

High-throughput RNA sequencing of the variegated and normal ‘Gonggan’ mandarin was performed using the Illumina HiSeq platform, yielding a total of 63.75 Gb clean data. After removal of low-quality reads and adapter sequences, 54,239,968, 54,808,660, and 54,298,836 clean reads were obtained from the normal ‘Gonggan’ mandarin samples CK1, CK2, and CK3, respectively (**Table 3**). For the variegated ‘Gonggan’ mandarin samples Var1, Var2, and Var3, the counts were 46,741,092, 56,247,346, and 56,826,062, respectively. The GC content of all samples was approximately 44%. The Q20 and Q30 values exceeded 96% and 90%, respectively, with a base error rate of 0.03% (below the 0.05% threshold). The mapping efficiency to the sweet orange reference genome was above 86% for all samples. In addition, the correlation coefficients among biological replicates of each sample were all greater than 0.91, indicating that the sequencing data was of high quality and suitable for subsequent analyses.

**Table 3:** Quality analysis and alignment statistics of transcriptome sequencing data.

Sample	Clean bases G	Clean reads	Error rate %	Q20 %	Q30 %	GC content %	Total mapped %	Uniquely mapped %	Multiple mapped %
CK1	8.14	54239968	0.03	97.54	93.14	44.71	89.51	83.93	5.58
CK2	8.22	54808660	0.03	97.40	92.85	44.34	89.39	84.41	4.98
CK3	8.14	54298836	0.03	97.09	92.13	44.24	89.55	84.63	4.92
Var1	7.01	46741092	0.03	97.45	92.95	44.78	89.93	84.93	5.00
Var2	8.44	56247346	0.03	96.60	90.86	44.76	89.52	84.52	5.00
Var3	8.52	56826062	0.03	97.23	92.42	44.61	89.97	84.99	4.98

CK, normal ‘Gonggan’ mandarin; Var, variegated ‘Gonggan’ mandarin.

#### 3.3.2 Identification of DEGs

Transcriptome data from the young fruit pericarp of variegated and normal ‘Gonggan’ mandarin was statistically analyzed. Using |log_2_(Fold Change)| > 1 and FDR < 0.05 as the screening criteria, a total of 1,438 DEGs were identified. Compared with normal ‘Gonggan’ mandarin, 950 genes were significantly upregulated and 488 genes were downregulated in the young fruit pericarp of variegated ‘Gonggan’ mandarin.

#### 3.3.3 GO functional enrichment analysis of DEGs

GO functional enrichment analysis was performed to investigate the functional classification of the DEGs. In total, DEGs were enriched in 1,144 GO terms, including 636 terms in the biological process (BP) category, 399 in the molecular function (MP) category, and 109 in the cellular component (CC) category (**Figure 2**). Specifically, the enriched BP terms were primarily associated with photosynthesis, oxidation-reduction process, single-organism metabolic process, protein folding, and metabolic process. In CC category, DEGs were primarily enriched in membrane protein complex, thylakoids, thylakoid parts, photosystems, photosynthetic membranes, and chloroplasts. Regarding MF category, dominant enrichments were detected in transition metal ion binding, cation binding, metal ion binding, oxidoreductase activity, and iron ion binding.

**Figure 2:**
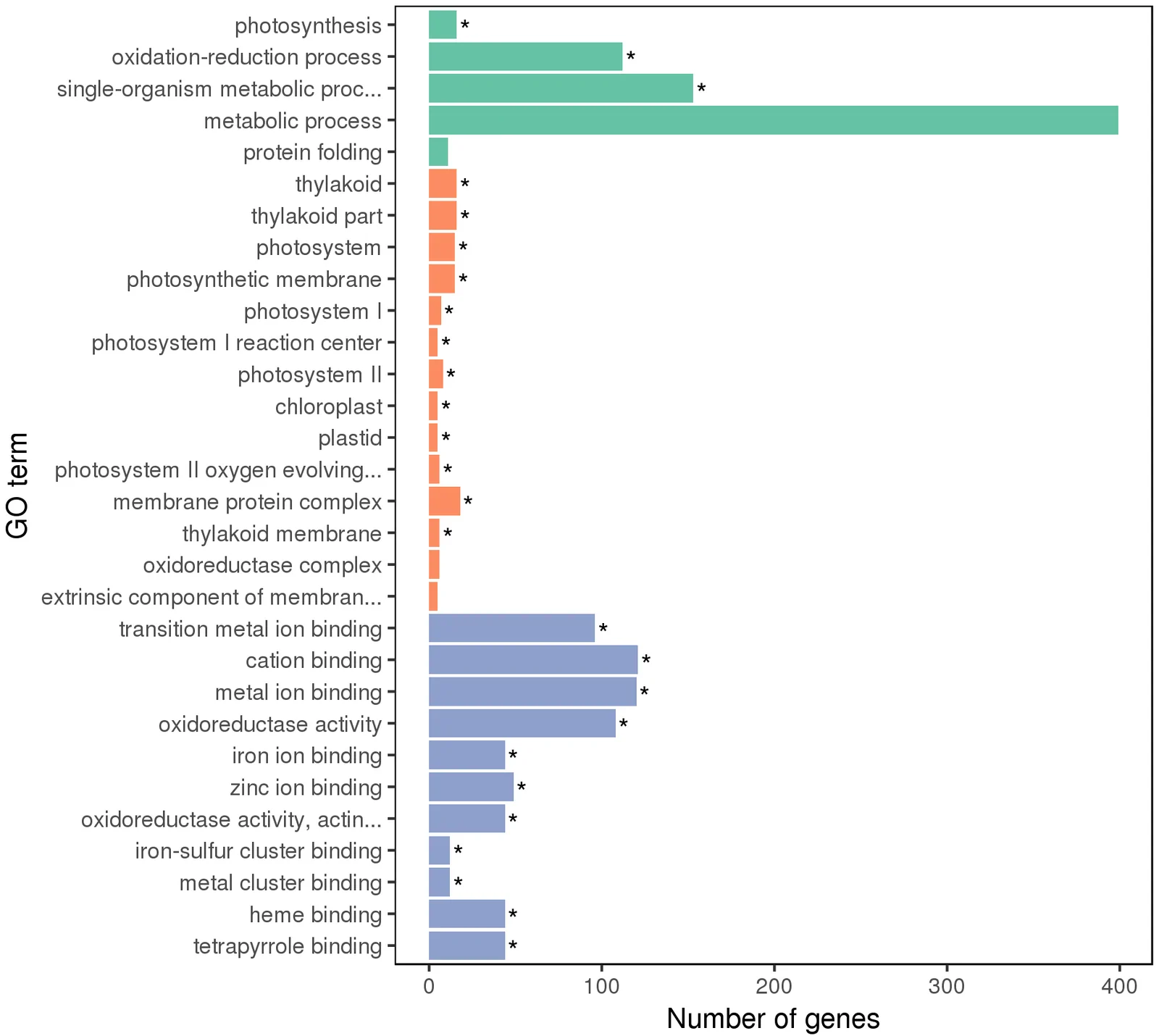
Bar chart of GO enrichment of DEGs. the abscissa indicated the GO term, and the ordinate indicated the corresponding GO term gene number., Green, biological process; Red, cellular component; Blue, molecular function. “*” indicates significantly enriched GO terms (padj < 0.05).

#### 3.3.4 KEGG pathway enrichment analysis of DEGs

KEGG pathway enrichment analysis was further conducted to identify the major biochemical and signaling pathways associated with DEGs. A total of 655 DEGs were successfully annotated and mapped to 103 pathways (**Figure 3**). Among these, 8 pathways were significantly enriched. Specifically, 136 DEGs (85 upregulated and 50 downregulated) were enriched in metabolic pathways; 80 DEGs (46 upregulated and 34 downregulated) in biosynthesis of secondary metabolites; 23 DEGs (all upregulated) in photosynthesis; 22 DEGs (14 upregulated and 8 downregulated) in biosynthesis of amino acids; 21 DEGs (14 upregulated and 7 downregulated) in carbon metabolism; 13 DEGs (6 upregulated and 7 downregulated) in glycolysis/gluconeogenesis; 10 DEGs (8 upregulated and 2 downregulated) in porphyrin and chlorophyll metabolism; and 6 DEGs (all upregulated) in photosynthesis-antenna proteins. These results suggested that the mottled pericarp of variegated ‘Gonggan’ mandarin was mainly associated with porphyrin and chlorophyll metabolism, as well as photosynthesis-related pathways.

**Figure 3:**
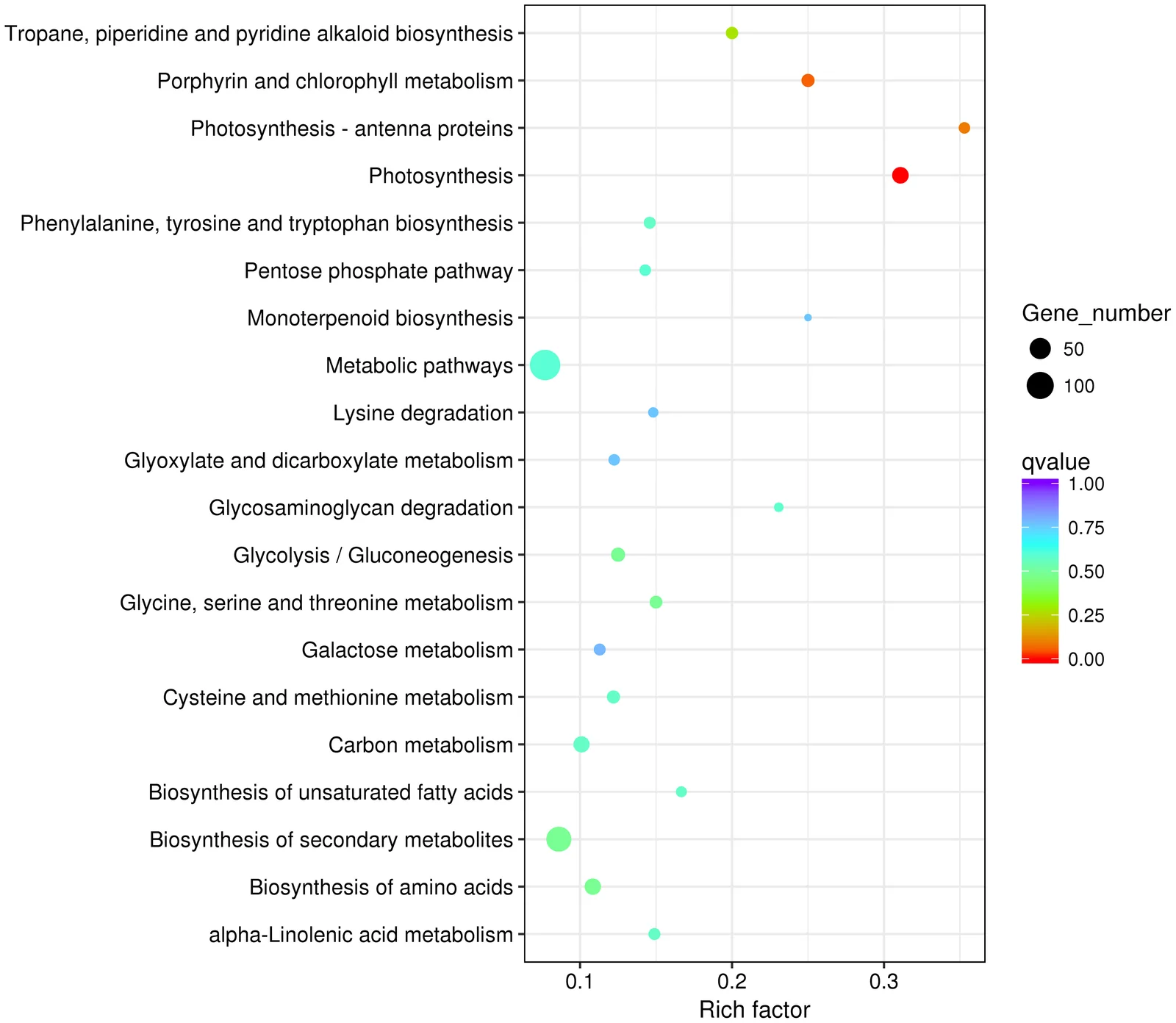
KEGG enrichment diagram of DEGs. the size of the circles indicated the gene count, and the color scale represented the q-value. The Rich factor (x-axis) reflected the proportion of genes enriched in each pathway.

#### 3.3.5 DEGs related to dysregulation of carotenoid and chlorophyll metabolic pathways

In carotenoid biosynthesis pathway, three key enzyme-coding genes, namely *DXS*, *CCD1* and *VDE1*, were significantly upregulated in the young fruit pericarp of variegated ‘Gonggan’ mandarin. Among them, 1-deoxy-D-xylulose-5-phosphate synthase (DXS) was the first and rate-limiting enzyme in the methylerythritol 4-phosphate (MEP) pathway, providing precursors for carotenoid biosynthesis. Compared with normal ‘Gonggan’ mandarin, the expression of the *DXS* gene Cs9g05150 was upregulated by 1.58-fold in variegated ‘Gonggan’ mandarin. Phytoene synthase (PSY) was the first rate-limiting enzyme in the carotenoid biosynthetic pathway, its encoding gene Cs6g15910 showed only a non-significant upregulation of 0.68-fold. By contrast, Cs5g26080, encoding violaxanthin de-epoxidase (VDE), and Cs7g01710, encoding carotenoid cleavage dioxygenase 1 (CCD1), were upregulated by 2.23-fold and 1.36-fold, respectively. The upregulation of these genes may have contributed to the enhanced carotenoid biosynthesis and metabolism in the young fruit pericarp of variegated ‘Gonggan’ mandarin.

Compared with normal ‘Gonggan’ mandarin, five core genes involved in chlorophyll biosynthesis were significantly upregulated in the young fruit pericarp of variegated ‘Gonggan’ mandarin (**Figure 4**). Among these, Cs3g16730, encoding glutamyl-tRNA reductase (HEMA), was upregulated by 1.21-fold. HEMA was the first enzyme and a key rate-limiting enzyme in the porphyrin biosynthesis pathway. Cs2g30990, encoding the magnesium chelatase subunit CHLD, was significantly upregulated by 1.34-fold. In addition, the transcript levels of Cs7g13850 (porphobilinogen deaminase), Cs6g16200 (Mg-protoporphyrin IX monomethyl ester cyclase), and Cs9g04920 (3,8-divinyl protochlorophyllide 8-vinyl reductase) were significantly elevated in variegated ‘Gonggan’ mandarin, reaching 1.75-, 1.80-, and 1.87-fold of those in normal ‘Gonggan’ mandarin, respectively.

**Figure 4:**
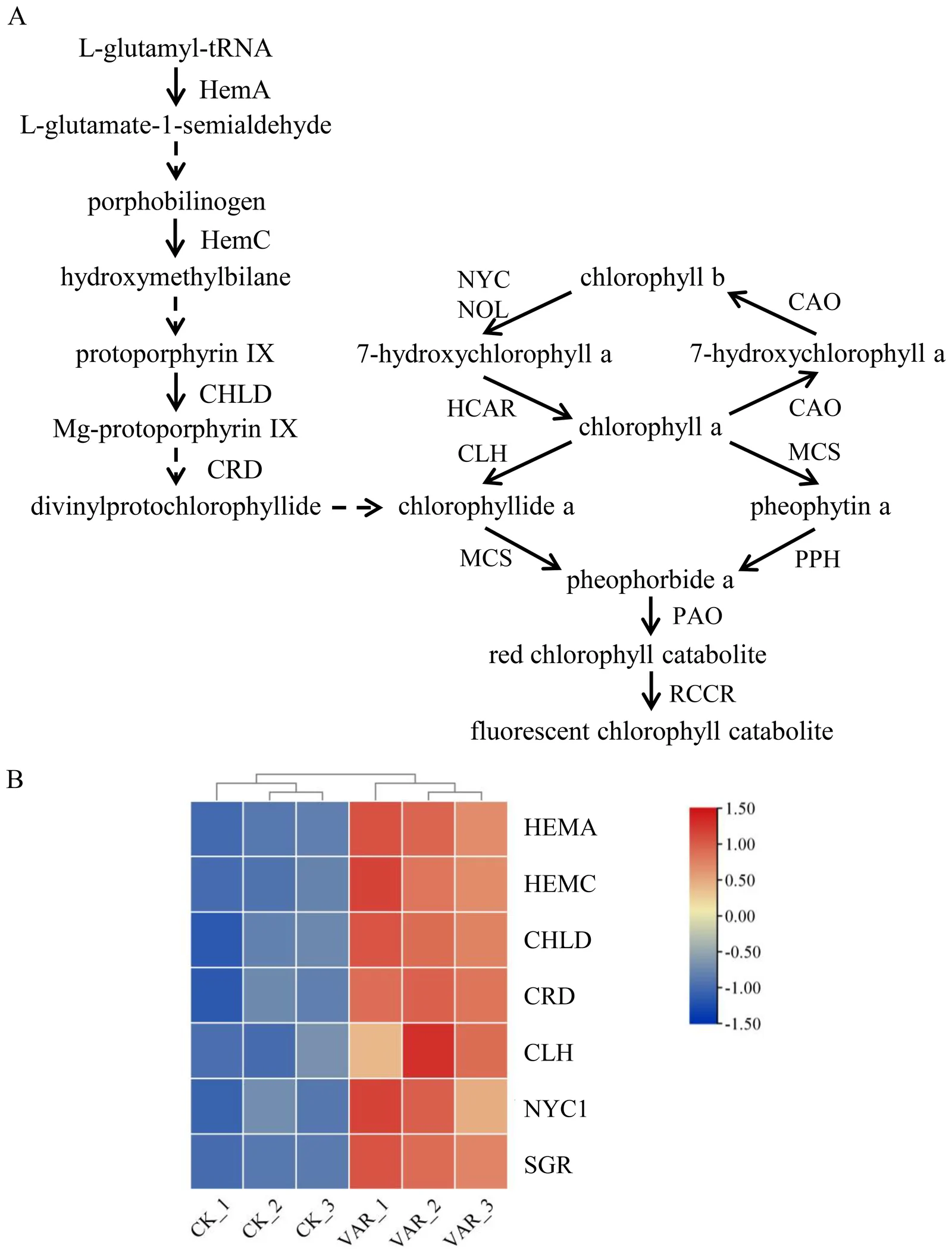
Schematic diagram of chlorophyll biosynthesis and degradation pathways highlighting DEGs in variegated ‘Gonggan’ mandarin. **(A)** Chlorophyll biosynthesis and degradation pathways. **(B)** Heatmap of DEGs in these pathways.

In terms of chlorophyll degradation, the expression of Cs4g15890, encoding chlorophyll b reductase, was significantly increased in the young fruit pericarp of variegated ‘Gonggan’ mandarin and was 1.28-fold that in normal fruit pericarp. Chlorophyllase was a vital rate-limiting enzyme in chlorophyll degradation, and its encoding gene, Cs9g07520, exhibited a 1.90-fold higher expression level in the variegated ‘Gonggan’ mandarin than in normal ‘Gonggan’ mandarin. Moreover, Cs3g20450, encoding the chloroplast degradation regulator stay-green 1 (SGR), was upregulated by 1.73-fold in variegated ‘Gonggan’ mandarin. Together, these expression changes indicated that both chlorophyll biosynthesis and degradation pathways might contribute to pericarp color formation in variegated ‘Gonggan’ mandarin.

#### 3.3.6 DEGs related to the photosynthetic system

Analysis of gene expression profiles in the young fruit pericarp of variegated ‘Gonggan’ mandarin showed that all 23 genes involved in the photosynthesis pathway were upregulated relative to normal ‘Gonggan’ mandarin (**Supplementary Table S1**). These DEGs encompassed key components of photosystem I (*PsaD*, *PsaE*, *PsaF*, *PsaG*, *PsaH*, *PsaK*, *PsaL*, and *PsaO*), photosystem II (*PsbO*, *PsbQ*, *PsbS*, *PsbW*, *PsbY*, *Psb27*, and *Psb28*), as well as the gamma subunit and b subunit genes of the F-type ATP synthase complex. In addition, six genes involved in the photosynthesis-antenna protein pathway were significantly upregulated in variegated ‘Gonggan’ mandarin. Among these, the light-harvesting complex I chlorophyll a/b-binding protein genes *Lhca1*, *Lhca2* and *Lhcb5* were upregulated by 2.31-, 2.30-, and 1.92-fold, respectively, while the light-harvesting complex II chlorophyll a/b-binding protein genes *Lhcb1*, *Lhcb4* and *Lhcb6* were significantly upregulated by 2.04-, 2.19-, and 2.33-fold, respectively. These genes were mainly involved in light energy capture and transfer. The coordinated upregulation of photosynthesis-related genes in variegated ‘Gonggan’ mandarin suggested that these transcriptional changes might be directly or indirectly associated with altered chlorophyll content in the mutant pericarp.

#### 3.3.7 DEGs involved in plant hormone signal transduction pathways

Further analysis of endogenous hormone-related DEGs identified 13 DEGs involved in four plant hormone signal transduction pathways (**Supplementary Table S1**). Among these, auxin-related genes were the most abundant, with five DEGs, followed by three ethylene-related genes and three abscisic acid-related genes.

In the auxin signaling pathway, two homologs encoding auxin/indole-3-acetic acid proteins (AUX/IAA), Cs1g13970 and Cs4g17050, were upregulated, whereas another homolog, Cs4g16220, was downregulated. In addition, two homologs encoding small auxin upregulated RNAs (SAURs), Cs8g08080 and orange1.1t04220, showed 2.49- and 1.77-fold higher expression levels, respectively, in the young fruit pericarp of variegated ‘Gonggan’ mandarin than in normal ‘Gonggan’ mandarin. These genes were involved in cell proliferation and plant growth and development. Regarding the ethylene signaling pathway, the ethylene receptor gene Cs8g12880 was upregulated, whereas Cs2g29100, encoding the transcription factor ethylene insensitive 3 (EIN3), and Cs5g29870, encoding an ethylene response factor (ERF), were downregulated by 1.20- and 2.63-fold, respectively, potentially affecting fruit ripening and senescence processes. In the abscisic acid signaling pathway, three homologous genes encoding protein phosphatase 2C (PP2C), namely Cs4g15360, Cs8g19140, and Cs9g16360, were significantly downregulated in the young fruit pericarp of variegated ‘Gonggan’ mandarin, with log_2_FC values of -3.58, -3.85, and -3.77, respectively. Finally, in the jasmonic acid signaling pathway, two DEGs, Cs7g31600, encoding coronatine-insensitive protein 1 (COI1), and orange1.1t00550, encoding a bHLH transcription factor MYC2, were downregulated by 1.23-fold and 1.73-fold, respectively. These genes were known to participate in the regulation of diverse developmental processes and stress-response processes.

## 4 Discussion

Fruit color, as a crucial external quality trait, makes an irreplaceable contribution to the sensory evaluation and commercial value of citrus fruit. Breeding novel citrus cultivars with bright coloration and high nutritional value represents a current trend in the domestic citrus industry (Viloria and Grosser, 2005; Liu et al., 2009; Zhu et al., 2021). Somatic variation breeding, particularly bud mutation selection, is one of the major approaches for cultivar improvement in perennial fruit crops, and many widely cultivated citrus cultivars worldwide have been derived through bud mutation selection (Wang et al., 2021, 2023). The variegated ‘Gonggan’ mandarin represents a valuable new citrus germplasm with both fresh consumption and ornamental potential. Exploring the physiological, biochemical and molecular mechanisms underlying its color variation is of great significance for the breeding of ornamental citrus cultivars.

Citrus fruit exhibits a wide range of pericarp colors, with immature fruit typically green and mature fruit predominantly orange-yellow, occasionally appearing purple-red or remaining green. Recent studies have shown that citrus pericarp coloration is mainly associated with the metabolism of chromatic pigments such as chlorophylls, carotenoids, and anthocyanins (Lu et al., 2025; Yang et al., 2025). Changes in the contents and relative proportions of these pigments were considered to be the major cause of fruit color variation. In ‘Zongcheng’ orange, the brownish pericarp coloration resulted from arrested chlorophyll degradation coupled with the accumulation of phytoene, phytofluene, and violaxanthin, creating a specific hue via the superimposition of persistent green and *de novo*-synthesized orange pigments (Zhu et al., 2021). ‘Qingougan’ was a stay-green mutant of ‘Ougan’, whose peel remained green at both the color-break and full-ripe stages. Pigment analysis revealed delayed chlorophyll degradation in the flavedo of ‘Qingougan’, with its total chlorophyll content reaching 4.3-fold higher than that of normal ‘Ougan’. In addition, α-carotene and β-carotene contents were significantly increased, whereas the levels of β-cryptoxanthin and violaxanthin were markedly reduced in ‘Qingougan’ compared with the normal ‘Ougan’ (Zhu et al., 2017). In this study, the contents of chlorophyll a and chlorophyll b in the young fruit pericarp of variegated ‘Gonggan’ mandarin decreased by 75.76% and 58.33%, respectively, whereas total carotenoid content increased by 28.61%. These results were highly consistent with the pericarp color parameter measurements, indicating that pigment composition was closely associated with pericarp color formation in the variegated ‘Gonggan’ mandarin.

In recent years, transcriptome sequencing has been widely used to study various aspects of citrus, such as growth, development, nutritional quality and stress response (Zhu et al., 2020; Rey et al., 2021; Khalilzadeh et al., 2022). In this study, transcriptome sequencing was performed on young fruits of the variegated ‘Gonggan’ mandarin to predict the molecular mechanism underlying its fruit pericarp color variation at the gene level. DEGs between the variegated ‘Gonggan’ mandarin and ‘Gonggan’ mandarin were mainly enriched in metabolic pathways, biosynthesis of secondary metabolites, and photosynthesis. Candidate genes potentially associated with the color variation of the variegated ‘Gonggan’ mandarin were preliminarily identified, which were primarily involved in chlorophyll degradation pathways.

Chlorophyll degradation is integral to leaf senescence and fruit ripening in plants (Guyer et al., 2014; Lado et al., 2019; Zhao et al., 2023). The genes encoding key enzymes in this catabolic pathway have been isolated and functionally characterized (Hörtensteiner, 2013; Zhang et al., 2014; Tanaka and Ito, 2025). Among them, chlorophyll b reductase, encoded by NYC1, was a short-chain dehydrogenase/reductase that catalyzes the conversion of chlorophyll b to 7-hydroxymethyl chlorophyll a, representing an early step in chlorophyll breakdown (Kusaba et al., 2007; Guan et al., 2022). Mutation of NYC1 in rice resulted in a stay-green phenotype during dark-induced senescence (Sato et al., 2009). Chlorophyllase was another key enzyme in the chlorophyll degradation pathway, hydrolyzing chlorophyll into chlorophyllide and phytol (Harpaz-Saad et al., 2007; Yin et al., 2016). Wang et al. (2017) found that chlorophyllase expression increased before fruit maturation in both ‘Huawan’ and seedless ‘Shatangju’, and then declined after maturity. Notably, the peak expression in ‘Huawan’ occurred 20 days later than that in the control, suggesting that chlorophyllase may contribute to maintaining the balance between chlorophyll synthesis and degradation during citrus fruit ripening. In addition, SGR1 was an important regulatory factor involved in chlorophyll degradation (Barry et al., 2008; Rong et al., 2013; Matsuda et al., 2016). Park et al. (2007) found that SGR1 interacted with LHCPII and formed complexes in the thylakoid membranes. In senescing leaves, SGR regulated chlorophyll degradation by inducing LHCPII disassembly through direct interaction, leading to the degradation of chlorophyll and chlorophyll-free LHCPII by catabolic enzymes and proteases, respectively. Disruption of the SGR gene in rice confers a stay-green phenotype during leaf senescence, whereas ectopic overexpressing of SGR produces yellowish-brown leaves. In *Arabidopsis*, SGR1 and SGR2 encoded Mg-dechelatases that specifically extracted magnesium from chlorophyll a and its associated protein complexes, thereby initiating the chlorophyll degradation pathway (Shimoda et al., 2016). Distinct from other plant species, two alleles of the SGR gene have been identified in citrus, namely SGRa and SGRb. Both can interact with PSY1, a rate-limiting enzyme in the carotenoid biosynthetic pathway, while only SGRa had chlorophyll degradation activity. In ‘Zongcheng’, a mutation in the coding region of SGRa introduced a premature stop codon, resulting in a truncated and non-functional protein. This led to impaired chlorophyll degradation and increased carotenoid accumulation in the peel, and the superposition of green and orange pigmentation ultimately resulted in the brown peel phenotype (Zhu et al., 2021). In the present study, key genes involved in chlorophyll degradation, namely chlorophyll b reductase, chlorophyllase, and SGR, were significantly upregulated in the pericarp of variegated ‘Gonggan’ mandarin, with expression levels 1.28-, 1.90-, and 1.73-fold higher than those in normal ‘Gonggan’ mandarin, respectively. These results indicated that chlorophyll degradation proceeded more rapidly in the young fruit pericarp of variegated ‘Gonggan’ mandarin, which was consistent with the lower chlorophyll content detected in pigment analysis. Therefore, we speculate that the reduced chlorophyll content in the young fruit pericarp of variegated ‘Gonggan’ mandarin is caused by the upregulation of chlorophyll degradation-related genes, including chlorophyll b reductase, chlorophyllase and SGR, which jointly accelerate chlorophyll degradation and ultimately reshape the pericarp color phenotype.

## 5 Conclusions

In this study, reduced chlorophyll content is a direct cause of the pericarp color variation in variegated ‘Gonggan’ mandarin. Transcriptome analysis identified multiple DEGs involved in chlorophyll biosynthesis and degradation, photosynthesis, photosynthesis-antenna proteins, and plant hormone signal transduction pathways. Among them, chlorophyll b reductase, chlorophyllase, and SGR1, which are key regulators in the chlorophyll degradation pathway, are closely associated with the change in chlorophyll content in the pericarp of variegated ‘Gonggan’ mandarin. These findings provide a theoretical basis for further studies on the mechanism underlying color variation in ‘Gonggan’ mandarin and support the potential utilization of the variegated ‘Gonggan’ mandarin cultivar as a novel ornamental and fresh-eating cultivar.

## Data Availability

Raw sequencing data have been submitted to the China National GeneBank DataBase (CNGBdb) under accession number CNP0010122.
